# The Role of Skin-to-Skin Contact and Breastfeeding in the First Hour Post Delivery in Reducing Excessive Weight Loss

**DOI:** 10.3390/children11020232

**Published:** 2024-02-10

**Authors:** Valentina Jurgelėnė, Vilma Kuzmickienė, Dalia Stonienė

**Affiliations:** 1 Department of Neonatology, Faculty of Medicine, Lithuanian University of Health Sciences, LT44307 Kaunas, Lithuania; valentina.jurgelene@lsmu.lt (V.J.); vilma.bimbiryte@stud.lsmu.lt (V.K.); 2 Department of Neonatology, Hospital of Lithuanian University of Health Sciences (LUHS) Kauno Klinikos, LT50161 Kaunas, Lithuania

**Keywords:** exclusive breastfeeding, skin-to-skin contact, neonate, excessive weight loss

## Abstract

Background and aims: An excessive weight loss (EWL) of >10% after birth is associated with serious health outcomes. The aim of this study was to determine factors that can reduce weight loss in full-term, exclusively breastfed infants after birth. Methods: This is a retrospective, observational, single-center study. We included 642 healthy, full-term, exclusively breastfed neonates born in 2019 in a baby-friendly hospital, and their healthy mothers. The exclusion criteria were as follows: supplementation with formula, multiple pregnancies, and neonates or mothers with health issues. Results: The mean percentage of neonatal weight loss after 24 h of life was 5.13%, and that after 48 h was 6.34%. Neonates delivered via a caesarean section lost more weight after 24 and 48 h of life than those delivered via vaginal delivery (*p* < 0.01). There is a noticeable pattern that neonates tend to lose more weight if they do not get skin-to-skin contact (SSC) and breastfeeding within the first hour after birth (*p* > 0.05). Conclusions: Neonates born via a CS tend to lose more weight after 24 and 48 h of life. Immediate SSC and breastfeeding in the first hour after delivery may decrease the excessive weight loss.

## 1. Introduction

Mother–newborn skin-to-skin contact (SSC) is a recommended practice worldwide [1,2]. During SSC, a bare infant is placed on a naked mother’s chest. SSC should be initiated as soon as possible after the delivery [1,2], and the mother and neonate should be warmly covered and left for at least an hour or until after the first breastfeed. It is fundamental to establish immediate and uninterrupted SSC in order to stimulate breastfeeding reflexes [1]. During vaginal delivery (VD), the fetal head is compressed and the fetus experiences intermittent hypoxia due to contractions; all those factors lead to catecholamine ascension in the neonatal blood instantly after birth. This results in olfactory, tactile, and thermal sensitivity, which makes the newborn crawl towards the nipple and establish a successful start to breastfeeding [3].

The World Health Organization (WHO), the United Nations Children’s Fund (UNICEF), the Centers for Disease Control and Prevention (CDC), and the American Academy of Pediatrics (AAP) recommend exclusively breastfeeding for the first six months of life [4]. This guidance is based on research that establishes a connection between breastfeeding and better health outcomes for infants, such as “lower occurrences of gastrointestinal infections, respiratory infections, otitis media, asthma, eczema, leukemia, obesity, sudden infant death syndrome (SIDS), and infant mortality”. The breastfeeding sufficiency is reflected by the neonatal daily weight during the first days of life [4].

Neonatal weight loss in the first days of life is a common physiological condition that affects almost all newborns [5]. A small amount of weight reduction due to postpartum fluid adjustments and active lipolysis is considered to be normal [6]. An excessive birth weight loss in the first week of life is associated with serious health outcomes—hyperbilirubinemia, hypoglycemia, dehydration—which can result in renal failure, thrombosis of small blood vessels (renal, cerebral, etc.), hypovolemic shock, and seizures [5,7,8]. The threshold of excessive weight loss differs worldwide and depends on the inner policy and chosen guidelines of the hospital. The American Academy of Pediatrics (AAP) suggests a threshold for full-term neonates of more than 7% [9], while the World Health Organization (WHO) and the National Institute for Health and Care Excellence of the United Kingdom propose more than 10% [10,11].

According to studies, one of the main risk factors for the excessive newborn weight loss is caesarean section (CS) delivery [5,8,12]. The latest research from the WHO shows that CS rates are rising globally [13]. In Lithuania, the birth rate via CS is 21.1% [14]. There are a few explanations of weight loss mechanisms in CS delivery: a delay in lactogenesis II and the excess of fluids administered to the mother during labor [12]. Due to these reasons, early intervention with formula supplementation may occur and thus decrease breastfeeding motivation [5,7].

If the physiological threshold of weight loss is exceeded, further assessment should be considered: neonatal clinical examination, observation of the breastfeeding, deciding on intervention to improve milk production, or adding another feeding method [12]. Most hospitals use formula supplementation to exclusively manage the weight loss of breastfed newborns, and formula supplementation may support weight gain, but often results in the cessation of breastfeeding [11,15]. Aspects that may have an impact on neonatal weight loss according to most studies are CS delivery, primiparous delivery, maternal age, and others. However, results are diverse and highly varied [5,8,12].

The aim of this study was to determine factors that can reduce weight loss in full-term, exclusively breastfed newborns.

## 2. Materials and Methods

### 2.1. Research Design

This is a retrospective, observational, single-center study. The data were collected from the hospital’s electronic medical record (EMR) system. The study design was used to determine factors that have an impact on minimizing neonatal weight loss in different delivery modes in order to improve neonatal health care. All data were de-identified.

### 2.2. Setting and Relevant Context

This retrospective study was conducted in a tertiary perinatal center (the Neonatology Department at the Hospital of Lithuanian University of Health Sciences (LUHS) Kauno klinikos), accredited as a baby-friendly hospital (BFH). The decision to add formula supplementation in our hospital is made when the weight loss threshold is more than 10% and lactation is insufficient. At the 7% threshold, close-up evaluation for possible breastfeeding problems is performed.

### 2.3. Sample

**Figure 1:**
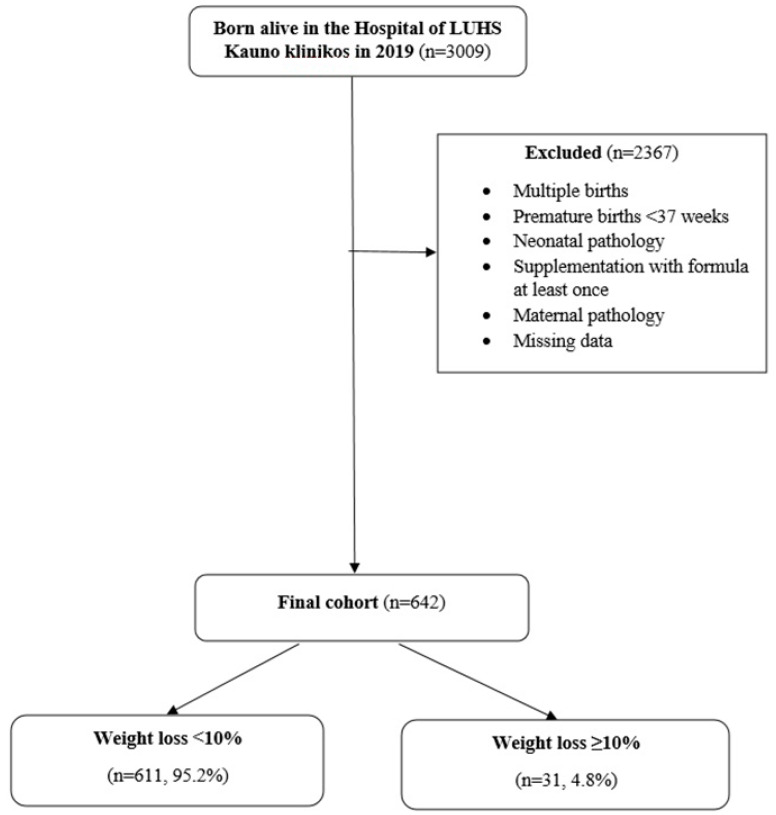
Cohort constitution.

We included 642 healthy, full-term (≥37 weeks), exclusively breastfed neonates born between 1 January 2019, and 31 December 2019, in the Hospital of LUHS Kauno klinikos and their healthy mothers (Figure 1). Multiple pregnancies, neonates with health issues (admitted to the neonatal intensive care unit (NICU), born with congenital anomalies), supplementation with formula at least once, and mothers with health issues during pregnancy and delivery met the exclusion criteria. Since the Hospital of LUHS Kauno klinikos is one of the perinatology centers in Lithuania, the vast majority of admitted women in labor have perinatal health issues, fetal pathologies, or complicated deliveries. This led to a great number of excluded participants. Only healthy full-term neonates and their healthy mothers were enrolled in order to avoid bias related to underlying diseases and an inability to breastfeed. The sample size was calculated using the target population formula with a confidence level of 99%.

### 2.4. Measurement

The demographic and perinatal data of participants were retrospectively obtained from the EMR. We assessed several neonatal (newborn sex, birth weight, breastfeeding in the first hour, SSC after birth) and maternal (gestational week at delivery, mode of delivery) variables. The sample size of emergency CS deliveries in our study was too small due to the exclusion criteria (maternal and neonatal pathologies); therefore, elective and emergency CS groups were merged. At our facility, SSC after a CS is performed immediately (within minutes) for stable mothers and their stable newborns, as soon as the mother is alert and responsive. The neonate is positioned onto the bare chest of the mother and is breastfed within the first hour of life. We collected the data of exclusively breastfeeding consecutive dyads in the first 72 h after birth. The weight was recorded in the first hour, more than 24 h, and more than 48 h after birth. All newborns were weighed with the same electronic scales. Weight reductions of ≥7% and an excessive weight loss (EWL) of ≥10% were evaluated.

### 2.5. Data Collection

Patients at the Hospital of LUHS Kauno klinikos give consent for the usage of their medical records for scientific purposes on admission day. In line with the retrospective nature of this study, the data of eligible neonate–mother dyads were collected from 1 November 2019 to 31 January 2020. De-identified data were recorded in Microsoft Excel (2013) and stored on a password-protected computer.

### 2.6. Data Analysis

For statistical analysis, data were exported from Microsoft Excel to SPSS (Version 29.0). To determine the means (±standard deviations) and percentages, descriptive statistics were used. The assumption of normality of a continuous variable was tested using the Kolmogorov–Smirnov test. Quantitative variables that met the condition of normality of distribution were calculated as the arithmetic mean and standard deviation V(SN); if they did not meet the condition, the median was used. To analyze two continuous variables, Student’s *t*-test or the Mann–Whitney U test was performed. The Kruskal–Wallis test or an ANOVA test were performed to analyze more than two continuous variables, as appropriate. For multiple pairwise comparisons, a posteriori Bonferroni test was used. Student’s paired *t*-test or the Wilcoxon test was used to compare the dependent variables. To analyze categorical variables, Pearson’s chi-squared (χ^2^) test was implemented. To assess the crude odds ratios and their 95% confidence intervals (CIs), univariate logistic regression analysis was performed. The multivariate logistic regression analysis was used subsequently. The difference was considered statistically significant when the significance level was *p* < 0.05.

## 3. Results

A total of 642 neonates and their mothers’ matching criteria were recruited in this study. The median of the maternal age was 31 [95% CI 30.5–31.3], the median parity was 2 [95% CI 2.1–2.3], the median of delivery was 2 [95% CI 1.8–1.9], and the median number of gestational weeks was 40 [95% CI 39.4–39.6]. A higher parity number (2 [95% CI 2.2–2.7], *p* = 0.06) and a shorter duration of pregnancy (39 [95% CI 39.1–39.5] gestational weeks, *p* = 0.019) were determined in the CS group (Table 1).

In the total population, 50.8% of neonates were male, and there were significantly more male infants in the CS group (*p* = 0.016). The neonatal birth weight median in the total population was 3540 [3509.6–3581.5] grams; it did not differ between VD and CS groups (*p* > 0.05). Apgar scores at 1 min and 5 min were higher in the VD group (*p* < 0.001). Immediate SSC was performed for 91.0% of all neonates. The rate of immediate SSC after birth was higher in the VD group—95.5% versus 65.6% (*p* < 0.001). Breastfeeding in the first hour of life was initiated in 89.6% of the total population. The initiation of breastfeeding in the VD group was 95.4% versus 48.6% in the CS group (*p* < 0.001) (Table 2).

The calculated probability that the neonate will not be breastfed in the first hour of life is higher if the newborn is male, the Apgar score after 1 min and after 5 min is less than 9, or the neonate is delivered via CS, with adjusted OR (95% CIs) of 1.7 [1.1–2.5], 2.2 [1.4–3.5], 2.8 [1.8–4.4], and 22.108 [11.9–41.2], respectively.

The mean percentage of neonatal weight loss in the total population after 24h of life was 5.13%, and that after 48h was 6.3%. Neonates delivered via a CS lost significantly more weight after 24 and 48 h of life than did those delivered via VD (*p* < 0.001) (Table 3). The probability of weight reduction ≥10% is higher after 24 and 48 h of life if the neonate is delivered via a CS, with adjusted odds ratios (95% [CI]) of 3.8 [1.0–14.6] and 4.5 [2.1–9.7] (Table 3).

**Figure 2:**
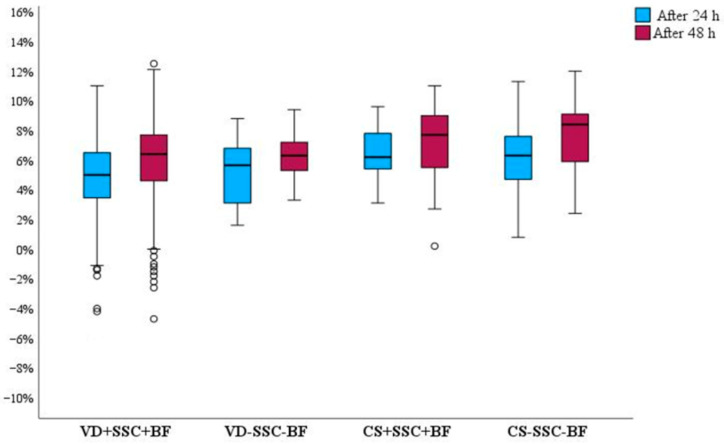
The impact of SSC and breastfeeding in the 1 h on the weight loss among neonates delivered via VD and CS. χ^2^ = 16.04, df = 3, *p* = 0.001 by Kruskal–Wallis test. Negative values in the Y axis means that neonates gained weight.

To compare the impact of immediate SSC and breastfeeding in the first hour after birth on EWL depending on delivery method, we formed a sample of four groups. The groups were divided by delivery mode. Two groups consisted of neonates who received immediate SSC and were breastfed in the first hour postpartum (groups were labeled VD+SSC+BF and CS+SSC+CS) (Table 4). The other two groups consisted of neonates who did not receive immediate SSC and were not breastfed in the first hour after delivery (VD-SSC-BF and CS-SSC-BF) (Figure 2).

The total sample size was 475 in the VD+SSC+BF group and 33 in the CS+SSC+BF group. After 24 h post delivery, 33.3% of neonates had lost >7% of weight in the CS* group, compared to 18.3% in the VD* group (*p* = 0.035). After 48 h postpartum, 63.3% of neonates had lost >7% of weight in the CS* group, compared to 39.6% in the VD* group (*p* = 0.007), and 9.1% of neonates had lost >10% of weight in the CS* group, compared to 2.7% in the VD* group (*p* = 0.043). Despite the initiation of SSC and breastfeeding in the first hour postpartum, neonates delivered via a CS tended to lose more weight than neonates delivered via VD (Table 4).

The median of weight loss in the VD+SSC+BF group was 5% after 24 h and 6.4% after 48 h. The median of weight loss in the VD-SSC-BF group was 5.7% after 24 h and 6.3% after 48 h. The median of weight loss in the CS+SSC+BF group was 6.2% after 24 h and 7.7% after 48 h. The median of weight loss in the CS-SSC-BF group was 7.6% after 24 h and 8.4% after 48 h. There is a noticeable pattern that neonates tend to lose more weight if they do not get SSC and breastfeeding in the first hour after birth (*p* > 0.05).

## 4. Discussion

There is an increasing amount of scientific research regarding the crucial benefits of timely SSC for mother–baby dyads. SSC after birth has a favorable effect on the newborn’s cardiorespiratory and thermoregulation stabilization, reducing the duration of crying, ensuring a successful start to breastfeeding and metabolic stability [2,16,17]. Immediate SSC has a positive effect on decreasing neonatal stress and blood cortisol concentration and helps to sustain blood glucose balance [18]. In addition to this, SSC postpartum has an impact on the lifelong health benefits, such as the formation of an adequate immune system response and the development of the microbiome, occurring via colonization with non-pathological bacterial flora from the mother, which increases brain development and improves behavioral outcomes [19].

Timely and uninterrupted SSC ensures an early beginning of breastfeeding, which provides the neonate with essential nourishment and sustained immunity [20]. Breastfeeding in the first hour postpartum ensures that the infant will receive colostrum. Colostrum, also known as “the first breastmilk”, is low in quantity but has a high concentration of bioactive substances [21]. Among the most vital components are antibodies that protect the newborn from possible infections, such as sepsis and pneumonia. Also, early breastfeeding reduces neonatal mortality rates from diarrhea, hypothermia, and mentioned infections [21,22,23].

Abdulghani et al., in their systematic review, described a few studies from the European region reflecting on SSC after vaginal birth rates [24]. A study from Croatia declared that 98% of mother–neonate dyads had SSC after VD. A national study in Denmark included over 200,000 births, and determined that 96% had SSC. Lower numbers were provided by Finland, Italy, France, and Spain—89%, 80%, 64%, and 29%, respectively. In our conducted study, immediate SSC was performed in 95.5% of vaginal births.

According to the studies, women who undergo a CS delivery and experience timely SSC postpartum have beneficial maternal and neonatal outcomes [19,21,25,26]. Firstly, this practice ensures comprehensive neonatal development, helps to form maternal–infant bonding, increases emotional well-being and confidence, and, most importantly, helps to establish a successful start to breastfeeding. However, despite the obvious benefits of SSC, separating the mother and infant is still a usual routine in many countries [24,25].

In A. Saxton’s et al. conducted cohort study, 57.5% of neonates had SSC with their mothers after a CS delivery. According to the results of the mentioned study, separation from the neonate after CS delivery and alack of timely SSC is a contributing aspect in breastfeeding difficulties [19,27]. It is determined that women experiencing a CS delivery have lower oxytocin and prolactin levels in blood compared to women that gave birth vaginally [28,29]. These hormones are fundamental in lactogenesis induction. A low hormonal concentration in blood is one of the risk factors in breastfeeding difficulties after a CS delivery. Timely SSC promotes oxytocin and prolactin production and lessens postpartum post-traumatic stress symptoms and postpartum depression (PPD) symptoms [29,30,31]. In our conducted research, 65.6% of the infants had SSC with their mothers after CS delivery in the operating room. However, these results do not reflect SSC in the entire CS delivery population, since neonates who had formula supplementation and mothers and newborns with health issues were excluded. Further studies should be performed. Also, as mentioned, mothers after CS deliveries are at a higher risk of experiencing depressive symptoms. We did not evaluate the psychological state of the mothers; therefore, we cannot confirm an association between a lack of SSC and PPD.

According to studies, a lack of SSC can have a negative impact on the neonatal state [2,28]. For instance, mothers experiencing PPD symptoms may have breastfeeding issues, and the psycho-emotional mother–baby bond may be damaged, leading to the child’s negative physical and psychological outcomes. About 6.5% to 20% of women experience PPD, and 20% to 40% experience postpartum depressive symptoms [32,33]. The essential difference between PPD and baby blues is that PPD lasts longer and has more severe symptoms that interfere with daily activities. In order to detect early symptoms of PPD, the Edinburgh postnatal depression scale (EPDS) was created [32,33]. Mothers fill in a questionnaire about their daily routine, and those who score above 13 are likely to have PPD [34]. Mothers suffering from PPD should be evaluated by a healthcare professional to confirm diagnosis and start timely treatment.

Dhanya Jayaraj et al. determined that higher maternal EPDS, lower latch, audible swallowing, mother’s nipple type, comfort and amount of help (LATCH) scores, and a lack of SSC lead to excessive weight loss [8]. The LATCH scoring system was developed in order to help healthcare professionals identify mothers with breastfeeding issues and ensure timely breastfeeding counselling before the discharge. This tool is easy to use; each of the five components is evaluated on a scale from 0 to 2, with the maximum score of 10 points. Shah et al. calculated that a LATCH score of ≥6 at discharge has the highest sensitivity (92.1%) and specificity (66.7%) for predicting exclusive breastfeeding at 6 weeks postpartum. Better LATCH scores are associated with longer breastfeeding, which improves the health of the infant [35]. Our facility does not use the LATCH score system for breastfeeding evaluation. The implementation of this tool in a daily practice would be helpful to detect the risk of neonatal EWL early.

We determined that neonates delivered via a CS without SSC and breastfeeding in the first hour tended to lose weight the most. Although statistical significance was not established, other researchers discovered the association between immediate SSC and EWL after a CS delivery. Jayaraj et al. found that the absence of immediate SSC is associated with EWL in exclusively breastfed infants [8]. Mezzacappa et al. reported that a CS delivery predisposes weight loss of >8% [12]. Similar results were found by Miyoshi et al.; they concluded that primiparity, antepartum CS, and an older age of the mother were the risk factors for excessive weight reduction during the first days of life [5]. Samayam et al. also reported a significant difference in CS-born neonates [36].

The mean percentage of neonatal weight loss in our hospital after 48 h was 6.3%, and the rate of excessive weight reduction of >10% was 4.4%. Our study results met the mean percentage of weight loss reported in the literature [7]. However, in some studies, researchers provided controversial results. Miyoshi et al. observed a 9.4% weight decrease and a high rate of excessive weight reduction, at 41%; according to authors, the cause could be the delayed lactogenesis in Asians [5]. Jayaraj et al. showed even higher results: the mean percentage of weight loss was 12.87% [8]. Neonates born at >34 weeks of gestation were included in the mentioned study; this factor may distort the results.

Flaherman et al. recruited over 100.000 exclusively breastfed neonates in their study to create nomograms depicting early weight loss by hour [37]. They determined that there is a significant difference in early neonatal weight loss, depending on the delivery mode. According to the nomograms, neonates born via a CS tend to lose more weight in the first days of life, and their time for weight regain is longer compared with that of those born by VD [37].

Tagi et al., in their study, implemented nomograms in daily practice, and determined that the “mean postnatal first–second day weight losses for vaginal and caesarean deliveries were 3.06% versus 4.7% and 4.5%, versus 5.8%, respectively, and were significantly higher for babies born by caesarean section (*p* = 0.001)” [38]. Also, 89.4% of vaginal births and 89.2% of neonates delivered via a CS were exclusively breastfed when the nomograms were used, compared to 64.2% of newborns born via a CS applying a daily weight loss strategy. The authors concluded that “the use of the early weight loss nomograms will decrease the rate of formula supplementation” [38].

Our study also determined that neonates delivered via a CS tend to lose more weight in the first days of life. According to Giudicelli et al., excessive weight reduction is associated with a greater amount (at least 1500 mL) of solute received during labor (*p*  < 0.001) [39]. It is known that fluids cross the placental barrier, primarily via simple diffusion, moving along an osmotic gradient. This can explain why fluids administered to women in labor may increase the extracellular fluid volume in the fetus. In the first week, the weight of full-term newborns may reduce by 5–7% of their birth weight [40]. Since the weight loss during the first days of life occurs due to the contraction of extracellular water, weight reduction may exceed the expected neonate body mass. On the contrary, Eltonsy et al. reported no significant relationship between intravenous fluids given to mothers during a CS and the increased risk of EWL [41]. Further investigation in that field should be performed.

Another explanation for excessive weight reduction and CS association is delayed lactogenesis II (DOLII), which is secretory activation that begins with copious milk formation after delivery [6,12,15]. DOLII appears in approximately one-third of women that have undergone a CS delivery [42]. Miyoshi et al. determined that intrapartum CSs did not affect neonatal weight; however, antepartum CSs were identified as an excessive weight loss (EWL) risk factor due to DOLII [5]. It is challenging to compare our study results accurately, since we did not distinguish the CS groups by urgency.

In the conducted study, we aimed to determine factors that can reduce neonatal weight loss in full-term, exclusively breastfed infants. The main motivation was to reduce the redundant consumption of formula supplementation in the first days of life. Chantry CJ et al., in their study, reported that early breast milk substitute supplementation increases the risk twofold in combined feeding (breastfeeding and formula supplementation) by day 30–60 of life. The risk was nearly triple in the cessation of breastfeeding by day sixty [43]. Unnecessary supplementation with formula alters the neonatal gut microbiome, makes newborns more susceptible to infectious diseases, and increases the risk of autoimmune disorders and allergies [44].

It is important for hospitals to enhance their daily postpartum care of the mother and neonate. Firstly, uninterrupted SSC must be a priority in the CS delivery group. Agudelo et al., in a randomized clinical trial, showed no significant differences between immediate and early SSC in exclusive breastfeeding at six months. These results indicate that if immediate SSC after a CS delivery is not possible, early SSC (within an hour) should be approached [45]. In another study, researchers established the significance of the SSC duration. The authors compared the control group to three SSC intervention groups: 30, 60, and 90 min of immediate SSC after a CS. In the prolonged SSC group of 90 min, 71% of neonates were exclusively breastfed at discharge, in comparison with 57% in the control group [46]. These results strongly emphasize the importance of immediate and uninterrupted SSC at the successful start of breastfeeding, especially in CS delivery groups [2,46].

## 5. Limitations

We faced some limitations in our study. First of all, we did not evaluate the maternal mental state using the EPDS tool. There is also a lack of breastfeeding objectification as we did not use the LATCH scoring system. In addition, in the conducted study, we did not record the duration of SSC and its impact on breastfeeding. All neonates who had formula at least once were eliminated. This induced biases—the study did not reflect neonates with excessive weight loss who had formula supplementation.

## 6. Conclusions

Neonates born via a CS tend to lose more weight after 24 and 48 h of life. Immediate SSC and breastfeeding in the first hour after delivery may decrease excessive weight loss.

## Figures and Tables

**Table 1:** Comparison of maternal characteristics by mode of delivery.

Variable	Total Population, *n* = 642	Delivery Mode		*p*
		VD, *n* = 529	CS, *n* = 113	
Maternal age (years), Mdn [95% CI]	31 [30.5–31.3]	30.5 [30.4–31.2]	31 [30.6–32.5]	0.201
Parity, Mdn [95% CI]	2 [2.1–2.3]	2 [2–2.3]	2 [2.2–2.7]	0.006
Delivery, Mdn [95% CI]	2 [1.8–1.9]	2 [1.8–1.9]	2 [1.7–2.1]	0.395
Gestational weeks, Mdn [95% CI]	40 [39.4–39.6]	40 [39.4–39.6]	39 [39.1–39.5]	0.019

VD—vaginal delivery, CS—caesarean section, Mdn—median, CI—Confidence interval, *p*-value (for continuous variables calculated using Mann–Whitney test, for nominal using Chi-square).

**Table 2:** Comparison of neonatal characteristics by mode of delivery.

Variable	Total Population, *n* = 642	Delivery Mode		*p*
		VD, *n* = 529	CS, *n* = 113	
Male, % (*n*)	50.8 (326)	48.6 (257)	61.1 (69)	0.016 ^+^
Birth weight (g), Mdn [95% CI]	3540 [3509.6–3581.5]	3545 [3494.9–3573]	3540 [3508.6–3691.7]	0.169
Apgar score at 1 min., Mdn [95% CI]	9 [9.2–9.3] *	9 [9.2–9.4] **	9 [8.8–9.2] ***	<0.001
Apgar score at 5 min., Mdn [95% CI]	10 [9.8–10] *	10 [9.7–10] **	10 [9.4–9.7] ***	<0.001
SSC, % (*n*) ^	91.0 (548)	95.5 (489)	65.6 (59)	<0.001
Breastfed in 1 h, % (*n*) ^	89.6 (516)	95.4 (481)	48.6 (35)	<0.001

VD—vaginal delivery, CS—caesarean section, SSC—skin-to-skin contact, Mdn—median, CI—Confidence interval, *p*-value (for continuous variables calculated using Mann–Whitney test, *^,^ **^,^ *** *p* < 0.001 according to Wilcoxon test, ^+^ *p* according to the asymptotic χ^2^ test); SSC (*n*) ^—total sample 602, due to missing data (*n* = 40); Breastfed in 1 h (*n*)^—total sample is 576 due to missing data (*n* = 66).

**Table 3:** Weight loss after 24 h and 48 h.

Weight Loss	Total Population, *n* = 642	Delivery Mode		*p*	OR [95% CI]
		VD, *n* = 529	CS, *n* = 113		
**≥7%**					
After 24 h, % (*n*)	22.1 (142)	18.0 (95)	41.6 (47)	<0.001	3.3 [2.1–5.02]
After 48 h, % (*n*)	43.8 (281)	39.3 (208)	64.6 (73)	<0.001	2.8 [1.8–4.3]
**≥10%**					
After 24 h, % (*n*)	1.4 (9)	0.9 (5)	3.5 (4)	0.033	3.8 [1–14.6]
After 48 h, % (*n*)	4.4 (28)	2.8 (15)	11.5 (13)	<0.001	4.5 [2.1–9.7]

VD—vaginal delivery, CS—caesarean section, *p*-value according to the asymptotic χ^2^ test, OR—Odds Ratio, CI—Confidence interval.

**Table 4:** EWL loss associations in neonates with immediate SSC and breastfeeding in the first hour after birth delivered via VD and CS.0.

Weight Reduction	VD+SSC+BF *n* = 475	CS+SSC+BF *n* = 33	*p*	OR [95% CI]
**≥7%**				
After 24 h, % (*n*)	18.3 (87)	33.3 (11)	0.035	2.23 [1.04–4.77]
After 48 h, % (*n*)	39.6 (188)	63.6 (21)	0.007	2.67 [1.28–5.55]
**≥10%**				
After 24 h, % (*n*)	1.1 (5)	0 (0)	NS	
After 48 h, % (*n*)	2.7 (13)	9.1 (3)	0.043	3.55 [0.96–13.15]

Asymptomatic *p*-value, OR—Odds Ratio, CI—Confidence Interval, NS—no significant difference, VD+SSC+BF—neonates delivered via VD who had immediate SSC and were breastfed in the first hour postpartum. CS+SSC+BF—neonates delivered via CS who had immediate SSC and were breastfed in the first hour postpartum.

## Data Availability

The data presented in this study are publicly available.
